# Breast cancer detection using deep convolutional neural networks and support vector machines

**DOI:** 10.7717/peerj.6201

**Published:** 2019-01-28

**Authors:** Dina A. Ragab, Maha Sharkas, Stephen Marshall, Jinchang Ren

**Affiliations:** 1 Electronics and Communications Engineering Department, Arab Academy for Science, Technology, and Maritime Transport (AASTMT), Alexandria, Egypt; 2 Electronic & Electrical Engineering Department, University of Strathclyde, Glasgow, United Kingdom

**Keywords:** The deep convolutional neural network, The support vector machine, The computer aided detection

## Abstract

It is important to detect breast cancer as early as possible. In this manuscript, a new methodology for classifying breast cancer using deep learning and some segmentation techniques are introduced. A new computer aided detection (CAD) system is proposed for classifying benign and malignant mass tumors in breast mammography images. In this CAD system, two segmentation approaches are used. The first approach involves determining the region of interest (ROI) manually, while the second approach uses the technique of threshold and region based. The deep convolutional neural network (DCNN) is used for feature extraction. A well-known DCNN architecture named AlexNet is used and is fine-tuned to classify two classes instead of 1,000 classes. The last fully connected (fc) layer is connected to the support vector machine (SVM) classifier to obtain better accuracy. The results are obtained using the following publicly available datasets (1) the digital database for screening mammography (DDSM); and (2) the Curated Breast Imaging Subset of DDSM (CBIS-DDSM). Training on a large number of data gives high accuracy rate. Nevertheless, the biomedical datasets contain a relatively small number of samples due to limited patient volume. Accordingly, data augmentation is a method for increasing the size of the input data by generating new data from the original input data. There are many forms for the data augmentation; the one used here is the rotation. The accuracy of the new-trained DCNN architecture is 71.01% when cropping the ROI manually from the mammogram. The highest area under the curve (AUC) achieved was 0.88 (88%) for the samples obtained from both segmentation techniques. Moreover, when using the samples obtained from the CBIS-DDSM, the accuracy of the DCNN is increased to 73.6%. Consequently, the SVM accuracy becomes 87.2% with an AUC equaling to 0.94 (94%). This is the highest AUC value compared to previous work using the same conditions.

## Introduction

Breast cancer is one of the leading causes of death for women globally. According to the World Health Organization (WHO), the number of cancer cases expected in 2025 will be 19.3 million cases. In Egypt, cancer is an increasing problem and especially breast cancer.

**Figure 1:**
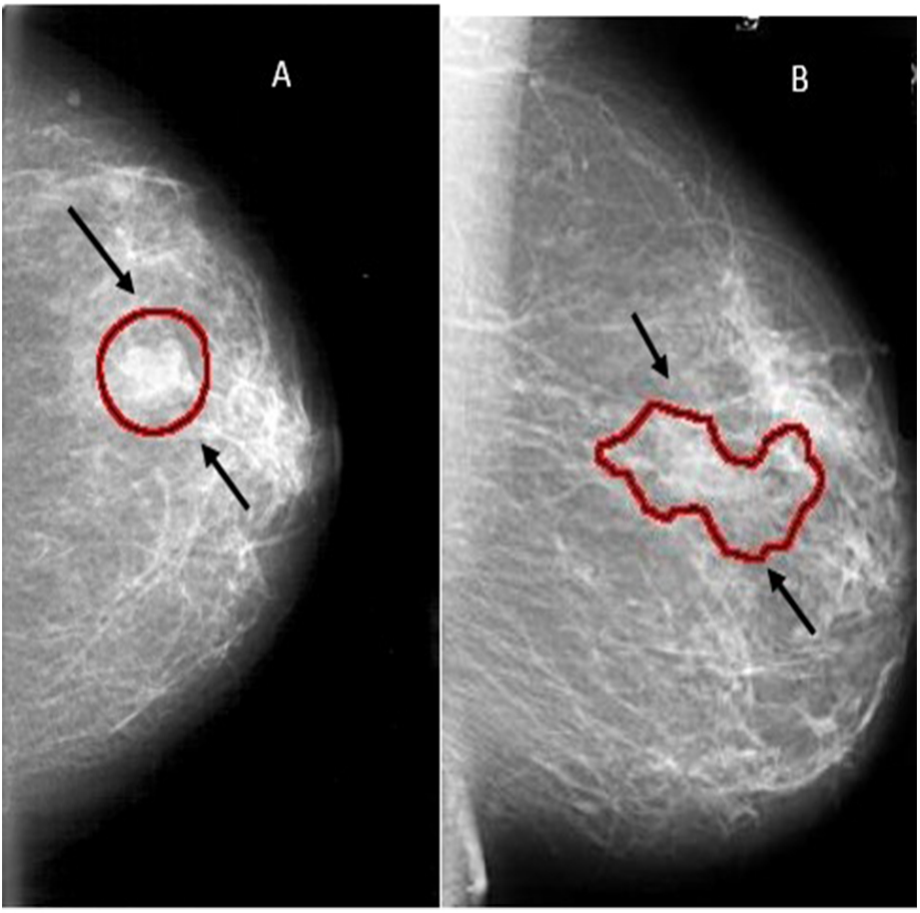
Examples of mammograms: (A) Mass ; (B) MCs.

Mammography is currently one of the important methods to detect breast cancer early. The magnetic resonance imaging (MRI) is the most attractive alternative to mammogram. However, the MRI test is done when the radiologists want to confirm about the existence of the tumor. The drawback of the MRI is that the patient could develop an allergic reaction to the contrasting agent, or that a skin infection could develop at the place of injection. It may cause claustrophobia. Masses and microcalcifcations (MCs) are two important early signs of the disease as shown in Fig. 1.

There are other indicators of breast cancer, such as architectural distortion (Bozek et al., 2009) but these are less significant.

A mass can be either benign or malignant. The difference between benign and malignant tumors is that the benign tumors have round or oval shapes, while malignant tumors have a partially rounded shape with an irregular outline. In addition, the malignant mass will appear whiter than any tissue surrounding it (Tang et al., 2009).

Recently, several researchers studied and proposed methods for breast mass classification in mammography images. Sharkas, Al-Sharkawy & Ragab (2011) used the discrete wavelet transform (DWT), the contourlet transform, and the principal component analysis (PCA) methods for feature extraction. The system was able to detect and classify normal and abnormal tissues. Additionally, it classified benign and malignant MC tumors. The achieved rate was almost 98%. Ragab, Sharkas & Al-sharkawy (2013) used the DWT as a feature extraction technique to detect mass abnormalities in the breast. In addition, a comparison between support vector machines (SVM) and artificial neural networks (ANN) for classifying normal, abnormal tissues, benign and malignant MCs tumors was introduced. The achieved detection rate was 96% for ANN and 98% for SVM (Ragab, Sharkas & Al-sharkawy, 2013). Cristina Juarez, Ponomaryov & Luis Sanchez (2006) applied the functions db2, db4, db8 and db16 of the Daubechies wavelets family to detect MCs. The achieved rate was close to 80% accuracy. Al-Sharkawy, Sharkas & Ragab (2012) detected mass lesions using the DWT and SVM, the rate achieved was 92%. Suzuki et al. (2016) used the deep convolutional neural network (DCNN) for mass detection. This study introduced the transfer learning in the DCNN. The sensitivity achieved when differentiating between mass and normal lesions was 89.9% using the digital database for screening mammography (DDSM) (Heath et al., 2001). Their study was the first demonstration for the DCNN mammographic CAD applications.

Dhungel, Carneiro & Bradley (2015) used the multi-scale belief network in detecting masses in mammograms. The sensitivity achieved was 85%–90% using the INbreast and DDSM-BCRP datasets, respectively. The main drawback of Dhungel, Carneiro & Bradley (2015) is the limited size of the training set. The number of training and testing used were 39 and 40 cases, respectively. Wichakam et al. (2016) used the DCNN and SVM. The sensitivity achieved was 98.44% using the INbreast dataset. Arbach, Stolpen & Reinhardt (2004) classified the MRI breast lesions using back propagation neural network (BPNN). They found that the area under the receiver operating characteristics (ROC) curve was 0.913. Sahiner et al. (1996) used the convolutional neural network (CNN) to classify normal and abnormal mass breast lesions. They used two segmentation techniques, the first technique employed averaging and subsampling. The second technique employed texture feature extraction methods applied to small sub-regions inside the ROI. The results obtained were 90% true positive rate (TPR) and 31% false positive rate (FPR). Jain & Levy (2016) used AlexNet to classify benign and malignant masses in mammograms of the DDSM dataset (Heath et al., 2001) and the accuracy achieved was 66%. Huynh & Giger (2016) used the DCNN features to classify benign and malignant tumors. The area under the curve (AUC) reached 0.81. Jiang (2017) introduced a new dataset named BCDR-F03 (Film Mammography dataset number 3). They used the GoogLeNet and the AlexNet, to classify breast lesions with an AUC of 0.88 and 0.83, respectively.

Zhu et al. (2017) proposed an end to end trained deep multi-instance networks for mass classification based on the whole mammogram image and not the region of interest (ROI).

Moreover, the deep learning methods were mentioned in some papers for breast cancer classification as in Dhungel, Carneiro & Bradley (2017a), Dhungel, Carneiro & Bradley (2017b), Dhungel, Carneiro & Bradley (2016), and Ching et al. (2017).

With reference to the literature, this manuscript presents a new CAD system to classify benign and malignant mass lesions from mammogram samples using deep learning based SVM. The main contribution is that two segmentation approaches are used: (1) segmenting the ROI manually and (2) using a threshold and region based techniques. The DCNN is used as the feature extraction tool whereas the last fully connected (fc) layer of the DCNN is connected to SVM to obtain better classification results. In addition, the experiments are tested on two datasets; (1) the DDSM and (2) the Curated Breast Imaging Subset of DDSM (CBIS-DDSM) (Lee et al., 2017).

## Methodology

Generally, a CAD system consists of several steps as follows (1) image enhancement, (2) image segmentation, (3) feature extraction, (4) feature classification, and finally, (5) an evaluation for the classifier.

**Figure 2:**
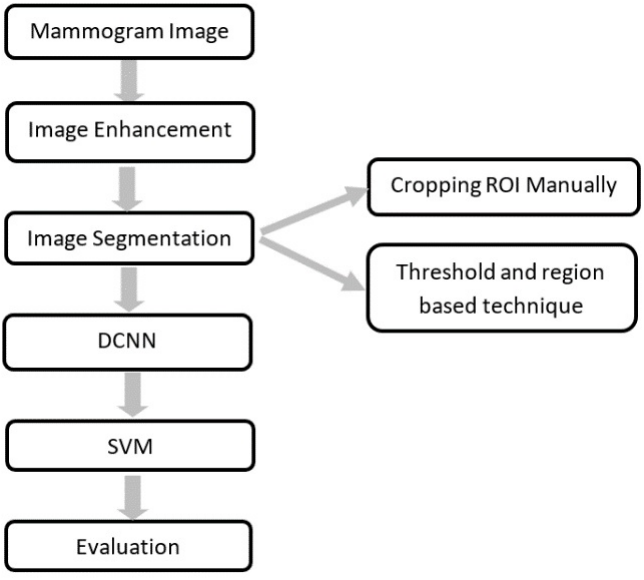
The block diagram of the CAD system.

The novelty of this work is to extract the ROI using two techniques and replace the last fully connected layer of the DCNN architecture with SVM. The proposed CAD system used in this work is illustrated in Fig. 2. Each block is described in detail in the following sub-sections.

### Image enhancement

Image enhancement is processing the mammogram images to increase contrast and suppress noise in order to aid radiologists in detecting the abnormalities.

There are many image enhancement techniques as in (Zabalza et al., 2015; Qiao et al., 2017) among which is the adaptive contrast enhancement (AHE). The AHE is capable of improving local contrast and bringing out more details in the image. It is an excellent contrast enhancement method for both natural and medical images (Pizer et al., 1987) and (Pisano et al., 1998). However, it can also produce significant noise.

In this manuscript, contrast-limited adaptive histogram equalization (CLAHE) which is a type of AHE will be used to improve the contrast in images (Pizer et al., 1987) and (Pisano et al., 1998).

One of the disadvantages of AHE is that it may over enhance the noise in the images due to the integration operation. Therefore, the CLAHE is employed as it uses a clip level to limit the local histogram in order to restrict the amount of contrast enhancement for each pixel (Sahakyan & Sarukhanyan, 2012).

The CLAHE algorithm can be summarized as follows: (Sahakyan & Sarukhanyan, 2012).

1. Divide the original image into contextual regions of equal size,
2. Apply the histogram equalization on each region,
3. Limit this histogram by the clip level,
4. Redistribute the clipped amount among the histogram, and
5. Obtain the enhanced pixel value by the histogram integration.

**Figure 3:**
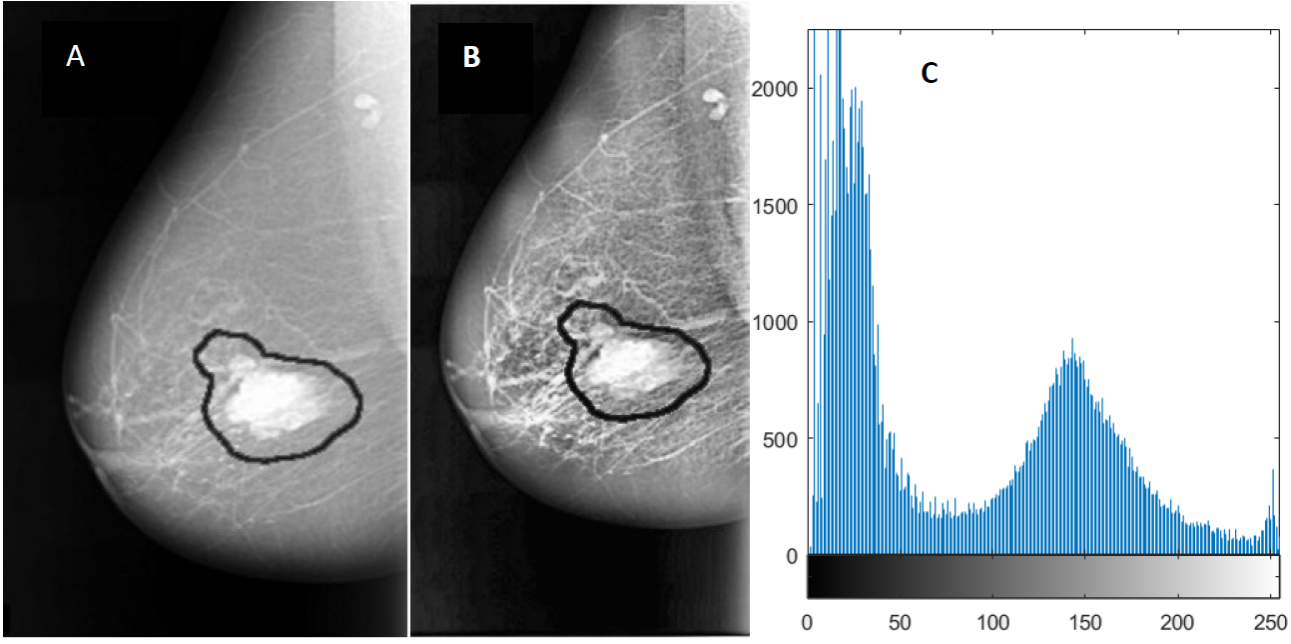
An example of image enhancement. (A) Original malignant mass case extracted from DDSM*,* (B) Enhanced image using CLAHE, and (C) Histogram representation of the image.

An enhanced image using CLAHE and its histogram representation is shown in Fig. 3.

### Image segmentation

Image segmentation is used to divide an image into parts having similar features and properties. The main aim of segmentation is to simplify the image by presenting in an easily analyzable way. Some of the most popular image segmentation methodologies are edge, fuzzy theory, partial differential equation (PDE), artificial neural network (ANN), threshold, and region-based segmentation (Kaur & Kaur, 2014).

#### Thresholding method

Thresholding methods are the simplest methods for image segmentation. The image pixels are divided with respect to their intensity level. The most common type of thresholding method is the global threshold (Kaur & Kaur, 2014). This is done by setting an appropriate threshold value (*T*). This value of (*T*) will be constant for the whole image. On the basis of (*T*) the output image *p*(*x*, *y*) can be obtained from the original image *q*(*x*, *y*) as given in Eq. (1),

(1) \begin{eqnarray*}p \left( x,y \right) = \left\{ \begin{array}{@{}l@{}} \displaystyle 1,ifq \left( x,y \right) \gt T \\ \displaystyle 0,ifq \left( x,y \right) \lt T \end{array} \right. \end{eqnarray*}

#### Region based segmentation method

The region-based segmentation is simpler than other methods. It divides the image into different regions based on predefined criteria (Khan, 2013). There are two main types for the region-based segmentation; (1) region growing and (2) region splitting and merging.

The region-growing algorithm has the ability to remove a region from an image based on some predefined criteria such as the intensity. Region growing is an approach to image segmentation in which neighbouring pixels are examined and joined to a region class where no edges are detected. It is also classified as a pixel-based image segmentation method as it involves the selection of initial seed point (Kaur & Goyal, 2015). It should be noted that the region splitting and merging method is the opposite of the region growing method as it works on the complete image (Kaur & Goyal, 2015).

**Figure 4:**
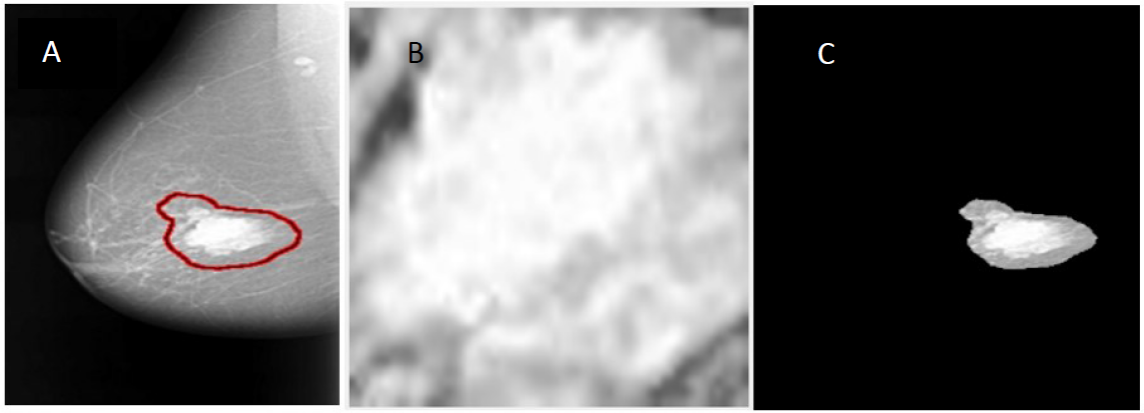
An example of image segmentation. (A) Original malignant mass case extracted from DDSM, (B) enhanced ROI extracted manually using circular contours, and (C) enhanced ROI extracted automatically by the region based method.

In this manuscript, the region of interest (ROI) is extracted from the original mammogram image by two different methods. Deep convolutional neural network The first method is to determine the ROI by using circular contours. The tumors in the DDSM dataset are labelled with a red contour and accordingly, these contours are determined manually by examining the pixel values of the tumor and using them to extract the region. (Duraisamy & Emperumal, 2017) cropped the ROI manually from the dataset. The ROI is shown in Fig. 4B.

In the second method, the threshold and the region-based methods are used to determine the ROI. The tumor in the samples of the DDSM dataset (Heath et al., 2001) is labelled by a red contour as illustrated in Fig. 4A. The first step to extract the ROI is to determine the tumor region by a threshold value, which is a value determined with respect to the red color pixel. After some trials, the threshold was set to 76 for all the images regardless of the size of the tumor. Then, the biggest area within this threshold along the image was determined and the tumor was cropped automatically. Figure 4C shows the ROI extracted by the threshold and the region based method. The steps for the used method can be summarized as follows:

1. Convert the original mammogram grayscale image into a binary image using the threshold technique.
2. Binary image objects are labelled and the number of pixels are counted. All binary objects are removed except for the largest one, which is the tumor with respect to the threshold. The largest area is the area enclosed within the red contour labelled around the tumor.
3. After the algorithm checks all pixels in the binary image, the largest area pixels within the threshold are set to “1”, otherwise all other pixels are set to “0”.
4. The resulting binary image is multiplied with the original mammogram image to get the final image without taking in consideration the rest of the breast region or any other artifacts.

### Feature extraction

There are many techniques for the feature extraction step. In recent years, deep convolutional neural networks (DCNN) have attracted great attention due to their outstanding performance. Consequently, in this manuscript the DCNN is used.

#### Deep convolutional neural network

DCNN has achieved success in image classification problems including image analysis as in (Han et al., 2015; Zabalza et al., 2016). A convolutional neural network (CNN) consists of multiple trainable stages stacked on top of each other, followed by a supervised classifier and sets of arrays named feature maps (LeCun, Kavukcuoglu & Farabet, 2010). There are three main types of layers used to build CNN architectures; (1) convolutional layer, (2) pooling layer, and (3) fully connected (fc) layer (Spanhol, 2016).

There are many CNN architectures such as CiFarNet (Krizhevsky, 2009; Roth et al., 2016), AlexNet (Krizhevsky, Sutskever & Hinton, 2012), GoogLeNet (Szegedy et al., 2015), the ResNet (Sun, 2016), VGG16, and VGG 19. However, the most commonly used architectures are the AlexNet, CiFarNet, and the Inception v1 (GoogleNet).

**Figure 5:**
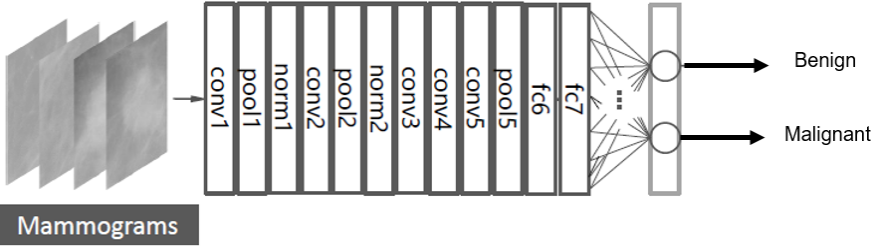
Fine tuning the AlexNet architecture.

The AlexNet architecture achieved significantly better performance over the other deep learning methods for ImageNet Large Scale Visual Recognition Challenge (ILSVRC) 2012. This success has revived the interest in CNNs in computer vision. AlexNet has five convolution layers, three pooling layers, and two fully connected layers with approximately 60 million free parameters (Krizhevsky, Sutskever & Hinton, 2012). The AlexNet CNN architecture is shown in Fig. 5.

The layers of conv1-5 in Fig. 5 are the convolution layers. Each neuron in the convolution layers computes a dot product between their weights and the local region that is connected to the input volume (Krizhevsky, Sutskever & Hinton, 2012).

The pooling layers are pool1, pool2, and pool5 as shown in Fig. 5. These layers perform a down sampling operation along the spatial dimensions to reduce the amount of computation and improve the robustness (Suzuki et al., 2016; Krizhevsky, Sutskever & Hinton, 2012).

The layers of norm1-2 in Fig. 5 are the normalization layers. They perform a kind of lateral inhibition that is observed in the brain (Krizhevsky, Sutskever & Hinton, 2012).

Additionally, the fully connected layers are fc6, fc7, and fc8 as shown in Fig. 5. Neurons in the fully connected layer have full connections to all neurons in the previous layer, as in ordinary feedforward neural networks (Krizhevsky, Sutskever & Hinton, 2012; Deng et al., 2009).

#### Transfer learning

The DCNN is pre-trained firstly using the ImageNet dataset, which contains 1.2 million natural images for classification of 1,000 classes. Then, the last fully connected layer is replaced by a new layer for the classification of two classes; benign and malignant masses. Figure 5 shows the fine-tuning of the AlexNet to classify only two classes (Deng et al., 2009).

To retrain the AlexNet after fine-tuning the fully connected layer to two classes, some parameters must be set; the iteration number and the primary learning rate are set to 10^4^ and 10^−3^, respectively. Whereas, the momentum is set to 0.9 and the weight decay is set to 5 × 10^−4^. These configurations are to ensure that the parameters are fine-tuned for medical breast cancer diagnosis. Other parameters are set to default values. The optimization algorithm used is the Stochastic Gradient Descent with Momentum (SGDM).

### Classification

In this step, the ROI is classified as either benign or malignant according to the features. There are lots of classifier techniques; such as linear discriminant analysis (LDA), artificial neural networks (ANN), binary decision tree, and support vector machines (SVM).

In this manuscript, the SVM is used because it achieved high classification rates in the breast cancer classification problem.

SVM is a machine learning algorithm that analyses data for classification and it is a supervised learning method that sorts data in categories. The aim of SVM is to formulate a computationally efficient way of learning by separating hyper planes in a high dimensional feature space (Gunn, 1998).

There are many hyper-planes that could classify two data sets. The optimum hyper-plane that should be chosen is the one with the maximum margin. The margin is defined as the width by which the boundary could increase before hitting a data point. The support vectors are considered the data points that the margin pushes up. Thus, the goal of the SVM is to find the optimum hyper-plane that separates clusters of target vectors on the opposing sides of the plane (El-naqa et al., 2002).

### Evaluation

There are several evaluation tools to assess a classifier amongst them, is the confusion matrix, the accuracy, the receiver-operating curve (ROC), the area under the ROC curve (AUC), the precision, and the F1 score.

#### The confusion matrix

The confusion matrix is a specific table visualizing the performance of the classifier.

Usually, in the field of machine learning a confusion matrix is known as the error matrix.

An image region is said to be positive or negative, depending on the data type. Furthermore, a decision for the detected result can be either correct (true) or incorrect (false). Therefore, the decision will be one of four possible categories: true positive (TP), true negative (TN), false positive (FP), and false negative (FN). The correct decision is the diagonal of the confusion matrix. Table 1 provides an example of the confusion matrix for two classes classification.

#### The accuracy

Accuracy is the measure of a correct prediction made by the classifier. It gives the ability of performance of the whole classifier. The accuracy is defined as in Eq. (2).

(2) \begin{eqnarray*}\text{accuracy}= \frac{TP+TN}{TN+FP+FN+TP} \end{eqnarray*}

#### The Receiver operating characteristic (ROC)

The ROC analysis is a well-known evaluation method for detecting tasks. Firstly, a ROC analysis was used in medical decision-making; consequently, it was used in medical imaging.

**Table 1:** A confusion matrix example.

**Class label**	**Predicted class label**	
	**Normal**	**Abnormal**
Normal	TN	FN
Abnormal	FP	TP

The ROC curve is a graph of operating points which can be considered as a plotting of the true positive rate (TPR) as a function of the false positive rate (FPR).

The TPR and the FPR are also called sensitivity (recall) and specificity, respectively. They are defined as in Eqs. (3) and (4).

(3) \begin{eqnarray*}\text{sensitivity}& = \frac{\mathrm{TP}}{\mathrm{TP}+\mathrm{FN}} \end{eqnarray*}

(4) \begin{eqnarray*}\text{specificity}& = \frac{TN}{TN+FP} \end{eqnarray*}

#### The Area under the ROC curve (AUC)

The AUC is used in the medical diagnosis system and it provides an approach for evaluating models based on the average of each point on the ROC curve. For a classifier performance the AUC score should be always between ‘0’ and ‘1’, the model with a higher AUC value gives a better classifier performance.

#### Precision

Precision is the ratio of correctly predicted positive observations to the total predicted positive observations. High precision relates to the low FPR. The precision is calculated using the following equation,

(5) \begin{eqnarray*}\text{Precision}= \frac{TP}{TP+FP} \end{eqnarray*}

#### F1 score

F1 score is the weighted average of precision and recall. It is used as a statistical measure to rate the performance of the classifier. Therefore, this score takes both false positives and false negatives into account. F1 score is defined as in Equation (6)

(6) \begin{eqnarray*}\mathrm{F}1 \text{score}= \frac{2\text{ * Recall * Precision}}{\text{Recall}+\text{Precision}} \end{eqnarray*}

### Experimental setup

The proposed DCNN based SVM classifier was applied to the mammogram images providing the possibility of each image to belong to one of the two classes either benign or malignant.

In this work, the most widely used DDSM mammogram dataset (Heath et al., 2001) has been chosen to verify the proposed methods using MATLAB. The DDSM dataset consists of 2,620 cases available in 43 volumes. The volumes could be normal, benign, or malignant samples. The resolution of a mammogram is 50 µm/pixel and the gray level depths are 12 bits and 16 bits.

Moreover, a new dataset is presented in this work, which is the Curated Breast Imaging Subset of DDSM (CBIS-DDSM) (Lee et al., 2017). It is an updated version of the DDSM providing easily accessible data and improved ROI segmentation. The dataset contains 753 microcalcification cases and 891 mass cases.

#### DCNN architecture

**Figure 6:**
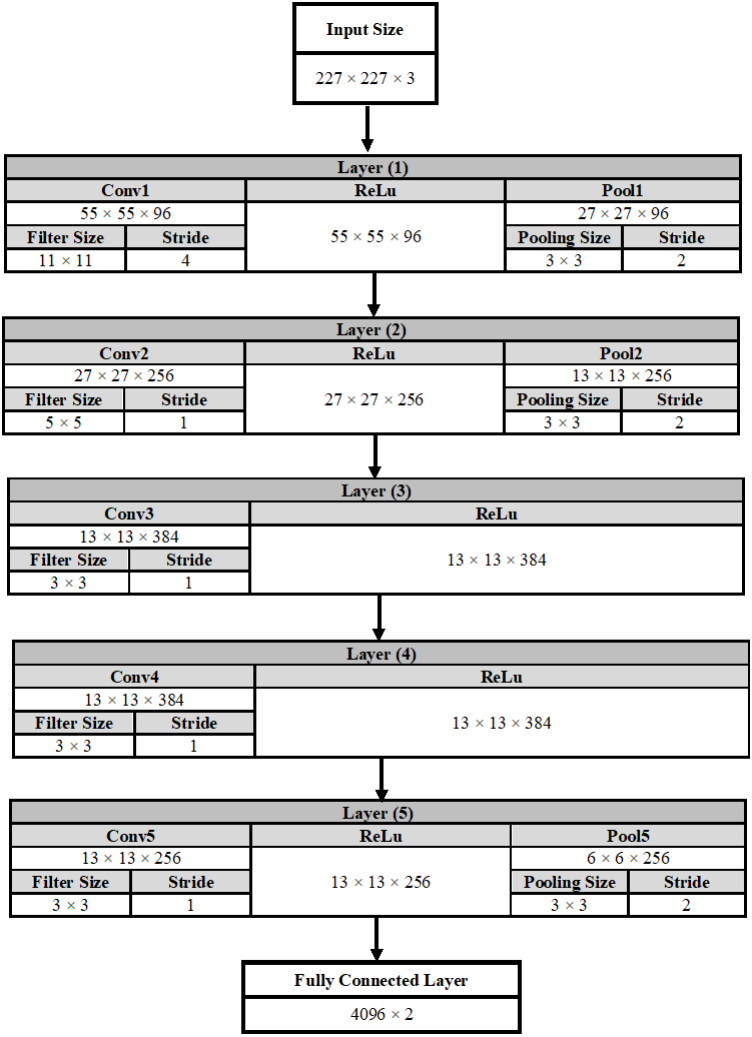
A detailed representation for AlexNet DCNN architecture.

The Alexnet DCNN architecture is used in this manuscript after fine-tuning to classify two classes instead of 1,000 classes. A conventional DCNN consists of a convolutional layer, a pooling layer, and a fully connected (fc) layer. The DCNN architecture is formed by stacking all these layers together. Figure 6 shows a complete description of each layer in the AlexNet architecture.

In the convolutional layer number (1) as an example, the output of this layer is calculated using Equation (7). The output is equals to 55 × 55 × 96, which indicates that the size of the feature map is 55 × 55 in width and in height. In addition, the number of feature maps is 96.

(7) \begin{eqnarray*}\text{The output size of the conv layer} = \left( \frac{input-filter size}{Stride} \right) +1.\end{eqnarray*}

On the other hand, the output size of the pooling layer is calculated using Eq. (8).

(8) \begin{eqnarray*}\text{The output size of the pool layer}= \left( \frac{output of conv-pool size}{Stride} \right) +1.\end{eqnarray*}

#### DCNN-based SVM Architecture

**Figure 7:**
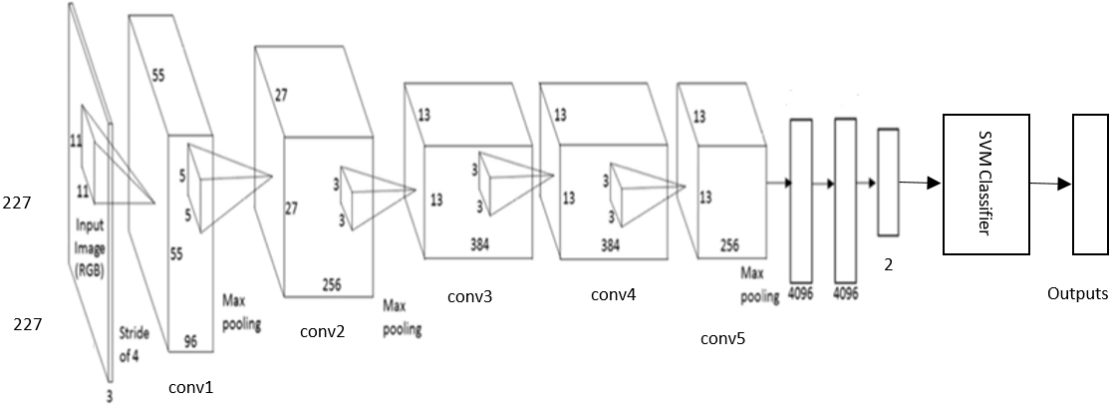
The DCNN –SVM fine-tuned AlexNet architecture.

The DCNN based SVM architecture is shown in Fig. 7. It consists of five stages of convolutional layers, ReLU activations, pooling layers, followed by three fully connected (fc) layers. The last fully connected layer is connected to SVM classifier to obtain better accuracy.

#### Data augmentation

Generally, training on a large number of training samples performs well and give high accuracy rate. However, the biomedical datasets contain a relatively small number of samples due to limited patient volume. Accordingly, data augmentation is a method for increasing the size of the input data by generating new data from the original input data. There are many strategies for data augmentation; the one used here in this manuscript is the rotation. Each original image is rotated by 0, 90, 180, and 270 degrees. Therefore, each image is augmented to four images.

## Results

### DDSM Dataset

A subset from the DDSM was extracted to apply the proposed methods. Each sample was augmented to four images. In this work 70% of images were used for training and the remainder for testing. This is the common ratio used in the classification problem. The number of training and testing samples for each segmentation technique is shown in Table 2. All experiments were validated using five cross fold validation.

**Table 2:** The number of training and testing samples for all the datasets used.

	**Training**	**Testing**	**Total**
DDSM (ROI cropped manually)	1,580	676	2,256
DDSM (ROI using threshold and region based)	1,288	552	1,840
CBIS-DDSM	3,691	1,581	5,272

First, the samples were enhanced and segmented using the two methods mentioned in ‘Methodology’. Then the features were extracted using CNN. The samples went through the SVM technique for classification.

To train the AlexNet, the maximum number of Epochs was set to 20.

The input layer of the AlexNet architecture requires that the size of the image is 227 × 227 × 3. Therefore, there is a pre-processing step to convert all the input images regardless of their sizes to the size required by the AlexNet.

**Figure 8:**
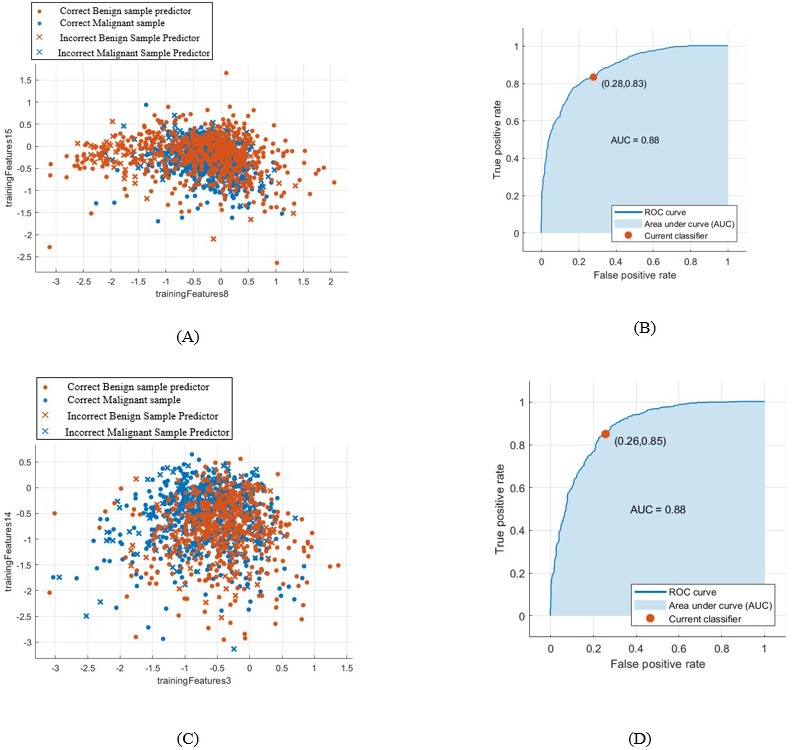
SVM classification between benign and malignant masses and the computed ROC for both segmentation techniques. (A) SVM classification between benign and malignant masses segmented by the first technique, (B) computed ROC for the first segmentation approach, (C) SVM classification between benign and malignant masses segmented by the second technique, and (D) computed ROC for the second segmentation approach.

When using the first segmentation technique the accuracy of the new-trained AlexNet was only 71.01%. This was achieved when extracting and classifying the lesions with the DCNN. Whereas, when attaching the DCNN to the SVM to obtain better result, the accuracy with linear kernel function was 79% with AUC equals to 0.88 (88%). Figure 8 (A) and (B) demonstrate the SVM classification accuracy between benign and malignant tumors samples and the ROC curve computed in this case.

Whereas, when using the second segmentation technique, the DCNN features accuracy reached only 69.2%. However, the accuracy of the SVM classifier with linear kernel function increased to 80.9% with AUC equals to 0.88 (88%). This is clear in Fig. 8C and in the computed ROC curve shown in Fig. 8D.

A comparison between all the SVM kernels with all the scores calculated for the two segmentation techniques are illustrated in Tables 3 and 4, respectively.

**Table 3:** The accuracy of SVM with different kernel functions for cropping the ROI manually for the DDSM dataset. Numbers in red indicate the best values between the several techniques.

**SVM kernel functions**	**Cropping ROI manually**					
	**Accuracy**	**AUC**	**Sensitivity**	**Specificity**	**Precision**	**F1 score**
Linear	**79%**	**0.88**	**0.763**	**0.822**	**0.85**	**0.8**
Quadratic	77.9%	0.87	0.764	0.797	0.81	0.786
Cubic	77.2%	0.86	0.759	0.781	0.79	0.774
Fine Gaussian	64.2%	0.74	0.741	0.598	0.43	0.544
Medium Gaussian	77%	0.87	0.754	0.787	0.8	0.776
Coarse Gaussian	73.7%	0.83	0.696	0.807	0.83	0.765

**Table 4:** The accuracy of SVM with different kernel functions for the threshold and region based technique for the DDSM dataset. Numbers in red indicate the best values between the several techniques.

**SVM Kernel functions**	**Threshold + region based segmentation technique**					
	**Accuracy**	**AUC**	**Sensitivity**	**Specificity**	**Precision**	**F1 score**
Linear	**80.5%**	**0.88**	**0.774**	**0.842**	**0.86**	**0.815**
Quadratic	80.1%	0.87	0.772	0.833	0.85	0.809
Cubic	78.3%	0.85	0.764	0.797	0.81	0.786
Fine Gaussian	54%	0.7	0.51	0.833	0.99	0.673
Medium Gaussian	79.1%	0.86	0.756	0.820	0.84	0.796
Coarse Gaussian	77.2%	0.85	0.736	0.813	0.84	0.785

When calculating the sensitivity, specificity, precision, and F1 score for each SVM kernel function for both segmentation techniques, it was proved that the kernel with highest accuracy has all the other scores high as well.

Furthermore, the testing error for the first and second segmentation techniques was 30.17% and 30.43%, respectively.

Table 5 summarizes all the results obtained for the classification of benign and malignant masses for both segmentation techniques for the DDSM dataset.

**Table 5:** The summary of the results obtained to classify benign and malignant masses for the DDSM dataset. Numbers in red indicate the best values between the several techniques.

	**Segmentation techniques**	
	**Cropping ROI manually**	**Threshold + Region based**
Trained DCNN accuracy	**71.01%**	69.2%
Error in testing	**30.17%**	30.43%
SVM accuracy	79%	**80.5%**
Sensitivity	**0.763**	0.774
Specificity	0.822	**0.842**
AUC	**0.88**	**0.88**
Precision	0.84	**0.86**
F1 score	0.8	**0.815**

#### CBIS-DDSM Dataset

For this dataset, the samples were only enhanced and the features were extracted using the DCNN. This is because that the samples of this dataset were already segmented.

**Figure 9:**
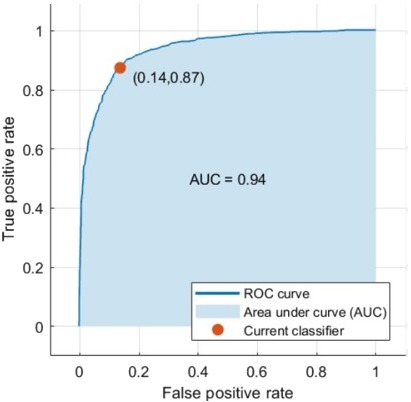
The computed ROC curve for the CBIS-DDSM dataset.

Data augmentation was applied to all the mass samples in this dataset as well to increase the training samples. The samples were augmented to four images using the rotation technique. When using the DCNN for feature extraction and classification the accuracy became 73.6%. Additionally, when classifying the features extracted from the DCNN using the SVM the accuracy with medium Gaussian kernel function reached 87.2% as illustrated in Table 6. The AUC was 0.94 (94%). The ROC curve is shown in Fig. 9.

By comparing to other researches results, either when using the AlexNet architecture with or other DCNN architectures, the results of the new proposed methods achieved the highest results. This is clear in Tables 7 and 8.

**Table 6:** Different evaluation scores calculated for SVM with different kernel functions for the CBIS-DDSM dataset. Numbers in red indicate the best values between the several techniques.

**SVM Kernel functions**	**CBIS-DDSM dataset**					
	**Accuracy**	**AUC**	**Sensitivity**	**Specificity**	**Precision**	**F1 score**
Linear	86.8%	0.94	0.854	0.876	0.88	0.866
Quadratic	85.6%	0.93	0.851	0.858	0.86	0.855
Cubic	84.6%	0.92	0.841	0.848	0.85	0.845
Fine Gaussian	69.9%	0.82	0.63	0.851	0.92	0.747
Medium Gaussian	**87.2%**	**0.94**	**0.862**	**0.877**	**0.88**	**0.871**
Coarse Gaussian	86.2%	0.93	0.854	0.876	0.88	0.866

**Table 7:** A comparative view of several mass detection methods based on AlexNet DCNN architecture, including the newly proposed method. Numbers in red indicate the best values between the several techniques.

**Reference**	**Contribution**	**Data set**	**AUC**	**Accuracy**
Jain & Levy (2016)	DCNN –**AlexNet** classify benign and malignant masses	DDSM	–	66%
Huynh & Giger (2016)	DCNN –**AlexNet** features to classify benign and malignant tumors	University of Chicago Medical Centre	**0.81**	–
Jiang (2017)	DCNN –**AlexNet**	BCDR-F03	**0.83**	–
The proposed CAD system	DCNN-SVM –**AlexNet** –cropping ROI manually –classify benign and malignant masses	DDSM	**0.88**	79.1%
	DCNN-SVM –**AlexNet** –threshold and region based –classify benign and malignant masses		0.88	80.9%
	DCNN-SVM –**AlexNet** –classify benign and malignant masses	CBIS-DDSM	**0.94**	**87.2%**

**Table 8:** A comparative view of several mass detection methods based on different DCNN architectures and datasets, including the newly proposed method. Numbers in red indicate the best values between the several techniques.

**Reference**	**Contribution**	**Data set**	**AUC**	**Accuracy**
Sahiner et al. (1996)	CNN to classify mass and normal breast	Dataset obtained by radiologists	0.87	–
Jain & Levy (2016)	DCNN—**AlexNet** classify benign and malignant masses	DDSM	–	66%
Huynh & Giger (2016)	DCNN—**AlexNet** features to classify benign and malignant tumors	University of Chicago Medical Centre	**0.81**	–
Jiang (2017)	DCNN—GoogLeNet DCNN –**AlexNet**	BCDR-F03	0.88 **0.83**	–
Duraisamy & Emperumal (2017)	DCNN –Vgg	MIAS and BCDR	0.9	85%
The proposed CAD system	DCNN-SVM –**AlexNet** –cropping ROI manually –classify benign and malignant masses	DDSM	**0.88**	79.1%
	DCNN-SVM –**AlexNet** –threshold and region based –classify benign and malignant masses		0.88	80.9%
	DCNN-SVM –**AlexNet** –classify benign and malignant masses	CBIS-DDSM	**0.94**	**87.2%**

In Table 7, some of the previous work using the AlexNet architecture is shown. On the other hand, Table 8 shows a comparative view of several mass detection methods based on DCNN, including the newly proposed method.

## Discussions

This work presented a new approach for classifying breast cancer tumors. It introduced a new CAD system including two approaches for segmentation techniques. The first one was cropping the ROI manually using circular contours from the DDSM dataset that was already labelled in the dataset. The second one depends on the threshold and region based techniques, the threshold was determined using the red contour surrounding the tumor area.

These two segmentation techniques were only applied on the DDSM dataset. However, for the CBIS-DDSM dataset the data provided was already segmented so therefore, no need for the segmentation step.

The features were extracted using the DCNN and especially the pre-trained architecture AlexNet. The transfer learning technique was presented by replacing the last fully connected layer with a new layer to differentiate between two classes; benign and malignant rather than 1,000 classes. The features went through the DCNN and SVM for classification, in which the last fully connected layer was connected to SVM to obtain better results.

To increase the number of training samples to improve the accuracy data augmentation was applied to the samples in which all the samples were rotated by four angles 0, 90, 180, and 270 degrees. This is demonstrated in Table 2.

For the DDSM samples, when using the DCNN as a classifier the accuracy of the new-trained architecture for the first segmentation method was higher than that of the second method. It recorded to be 71.01%.

For the DDSM samples when cropping the ROI manually, it is obvious from Table 3 that the SVM with linear kernel function achieved the highest values compared to the other kernels. The linear SVM achieved an accuracy of 79%and AUC, 0.88 (88%). Moreover, the sensitivity, specificity, precision, and F1 score reached 0.763 (76.3%), 0.822 (82.22%), 0.85 (85%), and 0.8 (80%), respectively which proved to be the highest values compared to the other kernels too.

Additionally, when using the threshold region based technique, the SVM with linear kernel function revealed to be the highest values compared to the others as well. This is clear in Table 4.

The accuracy, AUC, sensitivity, specificity, precision, and F1 score achieved 80.5%, 0.88 (88%), 0.774 (77.4%), 0.842 (84.2%), 0.86 (86%), and 0.815 (81.5%), respectively.

Therefore, when replacing the last fully connected layer of the DCNN by SVM to differentiate between benign and malignant masses, the accuracy for the region based method is higher than the manually cropped ROI method.

Additionally, the SVM with linear kernel function achieved the highest accuracy for both segmentation techniques compared to the other kernel functions.

Furthermore, the AUC for both segmentation methods were the same. One can easily notice this from the ROC curves shown in Figs. 8B and 8D of the first and second segmentation techniques, respectively.

Additionally, when testing the masses samples cropped manually and using the region based segmentation methods, 69.83% and 69.57% were correctly classified, respectively. This is clear in Table 5.

On the other hand, when using the CBIS-DDSM dataset, the samples were already segmented. Hence, the samples only went through the enhancement method using CLAHE and then the features were extracted using the DCNN.

Firstly, the features were classified using the DCNN, its accuracy increased to 73.6% compared to the DDSM samples. Whereas, when connecting the fully connected layer to the SVM to improve the accuracy, it yielded 87.2% accuracy with AUC equals to 0.94 (94%). This was clear in Fig. 9. This time the SVM with the Medium Gaussian achieved the highest values for all the scores compared to other kernel functions as demonstrated in Table 6.

The sensitivity, specificity, precision, and F1 score for the CBIS-DDSM dataset reached 0.862 (86.2%), 0.877 (87.7%), 0.88 (88%), and 0.871 (87.1%), respectively.

All the values achieved for the CBIS-DDSM were higher than that of the DDSM dataset, this is because that the data of the CBIS-DDSM were already segmented.

The error when testing the mass samples for the CBIS-DDSM dataset was 23.4%. This means that 76.6% from the total samples were correctly classified.

Finally, the proposed CAD system has been compared with other papers in the field that have the same conditions to prove the efficiency of the proposed method as shown in Tables 7 and 8.

Regarding the DCNN AlexNet architectures as in Table 7, the results have shown that, the proposed CAD system recorded the highest AUC, which was equal to 0.94 (94%) for the CBIS-DDSM dataset compared to Huynh & Giger (2016) and Jiang (2017). The former achieved AUC 0.81 (81%) while the latter achieved 0.83 (83%). Huynh & Giger (2016) applied their experiments on 219 breast lesions collected from the university of Chicago medical centre. The AlexNet with the transfer learning method was also used. However, Jiang (2017) used the dataset named BCDR-F03. They performed their tests on 736 mass cases. The ROI was extracted using Otsu segmentation algorithm. Besides, the transfer learning was used to classify two classes instead of 1,000 like in this manuscript.

When comparing with other researchers’ work with respect to using other DCNN architectures as illustrated in Table 8, the AUC achieved in this suggested work recorded the highest value as well.

## Conclusions

The goal of this work was to detect the masses and to classify benign and malignant tissues in mammograms.

A new CAD system was proposed. Two segmentation techniques were suggested. In the first technique, the ROI was cropped manually from the original image using circular contours. This was because the tumors in the DDSM dataset were labelled with a red contour.

Whereas, in the second technique, the region based method was used by setting a threshold, which was found to be equal to 76, and determining the largest area including this threshold.

In the feature extraction step, the DCNN was used. The AlexNet was retrained to distinguish between two classes and its parameters were changed to classify medical images.

The accuracy of the DCNN of the first segmentation method was higher than that of the second method by 1.8% using the DDSM dataset.

To achieve better accuracy, the last fully connected layer in the DCNN was replaced by the SVM.

When comparing between the two segmentation techniques for the DDSM dataset it was found that the SVM with linear kernel function for the second segmentation technique provided promising results. The accuracy, AUC, sensitivity, specificity, precision, and F1 score achieved 80.5%, 0.88 (88%), 0.774 (77.4%), 0.842 (84.2%), 0.86 (86%), and 0.815 (81.5%), respectively.

Moreover, when using the samples extracted from the CBIS-DDSM dataset, the accuracy of the DCNN increased to 73.6%. In addition the accuracy of the SVM with medium Gaussian kernel function became 87.2% with AUC reaching 0.94 (94%). Furthermore, the sensitivity, specificity, precision, and F1 score reached 0.862 (86.2%), 0.877 (87.7%), 0.88 (88%), and 0.871 (87.1%), respectively.

The proposed CAD system could be used to detect the other abnormalities in the breast such as MCs.

For future work, other networks will be suggested which include the very deep convolutional network (VGG) and the residual (ResNet) architecture.

## Additional Information and Declarations

### Data Availability

The following information was supplied regarding data availability: The results are obtained using the following publicly available datasets (1) the digital database for screening mammography (DDSM); and (2) the Curated Breast Imaging Subset of DDSM (CBIS-DDSM):
http://marathon.csee.usf.edu/Mammography/Database.html

https://wiki.cancerimagingarchive.net/display/Public/CBIS-DDSM.
